# The Effect of Glutathione Peroxidase-1 Knockout on Anticancer Drug Sensitivities and Reactive Oxygen Species in Haploid HAP-1 Cells

**DOI:** 10.3390/antiox9121300

**Published:** 2020-12-18

**Authors:** Steven Behnisch-Cornwell, Lisa Wolff, Patrick J. Bednarski

**Affiliations:** Institute of Pharmacy, University of Greifswald, 17489 Greifswald, Germany; steven.behnisch@uni-greifswald.de (S.B.-C.); lisa.wolff@uni-greifswald.de (L.W.)

**Keywords:** glutathione peroxidase-1, GPx1 knockout, anticancer drugs, reactive oxygen species

## Abstract

The role of glutathione peroxidases (GPx) in cancer and their influence on tumor prognosis and the development of anticancer drug resistance has been extensively and controversially discussed. The aim of this study was to evaluate the influence of GPx1 expression on anticancer drug cytotoxicity. For this purpose, a GPx1 knockout of the near-haploid human cancer cell line HAP-1 was generated and compared to the native cell line with regards to morphology, growth and metabolic rates, and oxidative stress defenses. Furthermore, the IC_50_ values of two peroxides and 16 widely used anticancer drugs were determined in both cell lines. Here we report that the knockout of GPx1 in HAP-1 cells has no significant effect on cell size, viability, growth and metabolic rates. Significant increases in the cytotoxic potency of hydrogen peroxide and *tert*-butylhydroperoxide, the anticancer drugs cisplatin and carboplatin as well as the alkylating agents lomustine and temozolomide were found. While a concentration dependent increases in intracellular reactive oxygen species (ROS) levels were observed for both HAP-1 cell lines treated with either cisplatin, lomustine or temozolamide, no significant enhancement in ROS levels was observed in the GPx1 knockout compared to the native cell line except at the highest concentration of temozolamide. On the other hand, a ca. 50% decrease in glutathione levels was noted in the GPx1 knockout relative to the native line, suggesting that factors other than ROS levels alone play a role in the increased cytotoxic activity of these drugs in the GPx1 knockout cells.

## 1. Introduction

In addition to catalase and superoxide dismutase, glutathione peroxidases (GPx) represent the first line defenses to protecting cells from oxidative stress and regulating cellular redox balance [1]. In the enzymatic reaction GPx reduces hydrogen peroxide and organic peroxides to water and corresponding alcohols, respectively [2]. Reducing equivalents come from two equivalents of glutathione (GSH), which are oxidized to glutathione disulfide (GSSG). Eight isoforms of GPx with varying occurrences in tissues and locations within the cell are known. Five isoforms utilize the rare amino acid selenocysteine in the active site, which increases the reactivity towards peroxides compared to their cysteine counterparts [3]. The most prevalent isoforms are GPx1 and GPx4 with ubiquitous localization in the cytosol/mitochondria and cell membranes, respectively. The nuclear GPx1 gene codes for both the cytosolic and mitochondria enzymes [4]. GPx1 degrades hydrogen peroxides while GPx4 is responsible for the removal of phospholipid peroxides in membranes [5]. It was found that a GPx1 knockout in mice has no fatal effects; offspring are even fertile, have a healthy appearance and express no special sensitivity against hyperoxia [6]. However, the formation of cataracts in the lens nucleus of GPx1 knockout mice as a result of oxidative damage has been observed [7]. In contrast, a knockout of GPx4 has lethal effects; fetuses die at an early state of embryogenesis and present with an intense phospholipid peroxidation in their cell membranes [8,9].

Glutathione peroxidases have been found associated with tumor progression, malfunction of anticancer therapy and development of drug resistance [5,10,11,12,13]. It is known that in some types of cancer GPx1 is upregulated, e.g., adult acute leukemia, thyroid tumors or urothelial carcinoma, whereas other types showing a downregulation such as adult chronic leukemia, breast cancer cell lines, gliomas or renal cell carcinoma [14,15,16,17,18,19]. Furthermore, it has also been reported that especially the upregulation of GPx in cancer tissues results in poor prognosis in tumor diseases such as kidney cancer, large cell and non-small cell lung carcinoma, squamous cell carcinoma or prostate cancer [20,21,22,23,24].

The goal of the current study was to evaluate the effect of GPx1 expression on cell viability and anticancer drug cytotoxicity. For these studies, the near-haploid HAP-1 cell line with a GPx1 knockout variant were obtained and characterized regarding various oxidative defense systems. The IC_50_ values of sixteen widely used anticancer drugs were determined in the native and GPx1 knockout variant, and the knockout index of every drug was calculated. The effects on reactive oxygen species (ROS) and GSH levels in both native and knockout cell lines were also evaluated.

## 2. Materials and Methods

### 2.1. Materials

The following materials were purchased from Sigma Aldrich (Taufkrichen, Germany): camptothecin, chlorambucil, colchicine, 5,5′-dithiobis(2-nitrobenzoic acid (DTNB), 2′,7′-dichlorofluorescine diacetate (DCF-DA), fetal bovine serum (FCS), glutathione (GSH), glutathione reductase, glucose 6-phosphate, glucose 6-phosphate dehydrogenase, melphalan, methotrexate, podophyllotixin, *tert*-butyl-hydroperoxide, thiotepa and vinblastine. IMDM medium stable glutamine and penicillin/streptomycin were from PAN Biotech (Aidenbach, Germany), 3-(4,5-dimethylthiazol-2-yl)-2,5-diphenyltetrazolium bromide (MTT) and paclitaxel were from Alfa Aesar (Haverhill, MA, USA), lomustine and temozolomide from Biomol (Hamburg, Germany), NADPH and p-nitrophenyl phosphate were from Carl Roth (Karlsruhe, Germany), cisplatin and carboplatin from Chempur (Karlsruhe, Germany), doxorubicin from Pharmacia & Upjohn (Stockholm, Sweden), oxaliplatin from Sanofi Aventis (Frankfurt a.M., Germany) and bortezomib from Selleckchem (Eching, Germany). All chemicals, kits and equipment for western blotting were obtained from Bio-Rad (Feldkirchen, Germany). The Annexin-V-FITC/propidium iodide kit was from Miltenyi Biotec (Teterow, Germany) and the ATP CellTiter-Glo kit was from Promega (Madison, WI, USA). For microtiter plate absorption measurements a Spectramax 384 Plus plate reader from Molecular Devices (Sunnyvale, CA, USA) was used. The antibody anti-GPx1 (#3206) was purchased from Cell Signaling (Cambridge, UK), anti-GPx4 (AB125066) from Abcam (Cambridge, UK), anti-Prdx-1 (LF-MA0214) from A-frontiers (Seoul, South Korea), anti-rabbit-HRP (A-6154) from Sigma Aldrich, whereas the antibodies for anti-Prdx-2, anti-Trx-1 and Trx-2 were produced and validated in the working group of Dr. Christopher H. Lillig (University Medicine, Greifswald; Institute of Medical Biochemistry and Molecular Biology, Greifswald, Germany) within and according to the following publication [25].

### 2.2. Cell Culture

The GPx1 knockout cell line (KO.HAP-1.GPx1, cat # HZGHC003261c1010) and its parental HAP-1 strain (cat # C631) were obtained under license from Horizon Discovery (Cambridge, UK). Cells were grown in IMDM medium supplemented with 10% FCS, 1% penicillin (10,000 U/mL) + streptomycin (10 mg/mL) and 1% stable glutamine in a humidified incubator with 5% CO_2_ atmosphere. Cells were transferred weekly into new culture flasks when confluence reached 75% (7500 cells in 5 mL medium into a T_25_ flask) to ensure growth in exponential phase. Once a week, the medium in culture was replaced by fresh one. Cell cultures were routinely tested for mycoplasma. Microscopy was performed with a DMi8 inverse microscope (Leica Microsystems, Wetzlar, Germany) fitted with an HC PL Fluotar L 20x/0.40 dry objective in a transmitted light phase contrast.

### 2.3. Determining Cell Sizes by Coulter Counter and EVE^TM^ Cell Counter

Two different methods were used to estimate the size of the HAP-1 and KO.HAP-1.GPx1 cells: changes in impedance with the Coulter Counter Z2 and image analysis with the EVE^TM^ counter. First, the cells were detached from the growth surface with 1.5 mL trypsin-EDTA solution and the reaction stopped with 3.5 mL complete growth medium. For measurements with the Coulter Counter Z2 (Beckman-Coulter GmbH, Krefeld, Germany), 100 µL of the cell suspension were added to 10 mL of Coulter Counter Isoton in triplicate. Each sample was measured and subsequently analyzed with help of the corresponding software Z2 AccuComp (ver. 3.01a). Three biological replicates with three technical replicates were analyzed and mean values calculated.

For measurements with the EVE^TM^ counter (NanoEnteck, Seoul, S. Korea), 10 µL of the cell suspension were diluted with 10 µL of phosphate buffered saline (PBS) and subsequently added to 10 µL of a Trypan blue solution. After mixing, 10 µL were transferred to measurement slides and inserted into the counter. Data analysis was performed with the corresponding software EvePC (ver 1.01). Mean values of both cell size and live/dead ratios were calculated from three biological replicates with three technical replicates each.

### 2.4. Determining Cell Doubling Times via Crystal Violet Assay

To determine growth rates and, subsequently, doubling times of both HAP-1 and KO.HAP-1.GPx1 cells the crystal violet cell proliferation assay was performed [26]. Cells were seeded at a density of 1000 cells per 0.1 mL medium per well and allowed to grow for 24, 48, 72 and 96 h, respectively. After each interval, cells were fixated by using 100 µL of a 1% glutaraldehyde in PBS solution and afterwards stored in 100 µL PBS until staining. Staining was performed by adding 100 µL of a 0.02% crystal violet in water solution for 20 min with subsequent washing by soaking the plates in cold water for 30 min. The crystal violet adheres to the DNA of cells present, excess dye is washed off and the remaining stain dissolved by adding 100 µL of 70% EtOH. Following, the plate optical densities (OD) were read at λ = 570 nm in a microplate reader and the growth rates determined according to Equation (1) and doubling time by Equation (2):

(1) $$\mathrm{growth}\;\mathrm{rate}\;\left(\mathrm{gr}\right)=\frac{\ln\left(\frac{N\left(t\right)}{N\left(0\right)}\right)}{t}$$

(2) $$\mathrm{doubling}\;\mathrm{time}=\frac{\ln\left(2\right)}{\mathrm{gr}}$$

(N(t) = OD at time t; N (0) = OD at time 0; t = time in h).

### 2.5. Assessing Rates of Cellular Metabolism with the APH and MTT Assays

To compare the metabolism of HAP-1 and the corresponding GPx1-knockout cell line, two different microplate-based assays were performed. Both include the conversion of a substrate into a photometrically detectable product by intracellular enzymes which may be used to represent the cellular metabolism. The rate of conversion was monitored by detecting the corresponding ODs of the products over a course of three and four hours, respectively.

The acidic phosphatase (APH) assay is based on the principle that intracellular APH hydrolyze p-nitrophenyl phosphate (PNPP) to p-nitrophenol (PNP). An increase of PNP can subsequently be detected at a wavelength of λ = 405 nm. Additionally, the MTT assay was used to assess whether there are any differences between the two cell lines’ metabolisms. The tetrazolium bromide compound (MTT), water soluble and of yellow color, will be reduced to the corresponding insoluble MTT-formazan product by mitochondrial dehydrogenases of viable cells. The presence of this purple product can be detected at λ = 570 nm.

For both the MTT and the APH assay, 20,000 cells were seeded in 0.1 mL medium per well of a 96-well plate and allowed to adhere for 24 h at standard culturing conditions (37 °C, 5% CO_2_, humidified atmosphere).

For the MTT assay, every 30 min 20 µL of a 2.5 mg/mL MTT in PBS solution were added to four wells per cell line over a time of four h. After the last interval, the supernatant was removed from the wells and 50 µL of DMSO added in order to dissolve the formazan crystals. OD measurement were performed at λ = 570 nm with a microplate reader.

For the APH assay, over a time of three hours every 20 min the medium was removed from four wells per cell line, the monolayer washed twice with 200 µL PBS and subsequently filled with 100 µL PBS and 100 µL of a 2.0 mg/mL PNPP in APH buffer solution. After the last interval 20 µL of 1 N NaOH was added per well and within 10 min the plate ODs measured at λ = 405 nm with a microplate reader. From the OD data, rates were calculated and, subsequently, the rate of the knockout cell line set in relation to that of the parental cell line to allow for comparison.

### 2.6. Assessing Cell Viability with the ATP Assay and Annexin-V Assays

This assay were performed to compare the basic viability in the HAP-1 and KO.HAP-1.GPx1 lines. The assay is based on the principle that luciferin is converted to oxyluciferin by the enzyme luciferase in the presence of ATP and oxygen. All components are added with the assay reagent except for the ATP which is provided by the cells. Thus, the resulting luminescence depends on the intracellular ATP content. The intracellular ATP level was measured by using the CellTiter-Glo^®^ Luminescent Cell Viability Assay following the corresponding protocol. Briefly, 20,000 cells were seeded in 0.1 mL per well of a 96-well plate and allowed to adhere overnight. Control wells with medium only were prepared. The assay reagent was prepared as described and 100 µL added per well. The plate was placed on a shaker for 2 min to support cell lysis. Afterwards, 10 min incubation time were added to stabilize the luminescent signal which was subsequently analyzed using a Tecan infinite 200PRO plate reader and the corresponding software Tecan i-control.

The Annexin-V-FITC/propidium iodide assay was performed according to the manufacturer’s instructions with some adaptations. The assay is based on the principle that fluorescent Annexin-V FITC can only bind to phosphatidylcholine on the outer leaf of the cell membrane during both early and late apoptosis while propidium iodide only enters cells and binds to DNA during late apoptosis. Briefly, 125,000 cells were seeded in 2 mL medium per well of a 6-well plate and incubated for 24 h under standard conditions. The medium was exchanged and the cells cultured for additional 24 h. Samples were then collected by trypsinization, centrifuged for 5 min at 500× *g* and the supernatant removed. The cell pellet was washed once with 500 µL Assay Binding Buffer and centrifuged again for 5 min at 500× *g*. After aspirating the supernatant the cells were resuspended in 50 µL of Assay Binding Buffer and 5 µL of Annexin V-FITC conjugate were added and incubated for 15 min at room temperature in the dark. Another washing step with 500 µL Assay Binding Buffer followed and finally the pellet was resuspended in 250 µL Assay Binding Buffer for measurement. Immediately before flow cytometric analysis, 2.5 µL of a 100 µg/mL propidium iodide solution was added.

### 2.7. Determination of Total Glutathione Content in Cell Lysates

For the determination of the total glutathione (GSH + GSSG) content of cell lysates, an enzymatic recycling assay was used, which is based on the oxidation of GSH to GSSH by Ellman’s reagent and the restauration of GSH by a NADPH/GR and G-6-P/G-6-P-DH system [27]. This method determines both the cellular concentration of GSH and GSSG together. All solutions were prepared in a phosphate buffer (50 mM, pH = 7.4) containing 1.1 mM EDTA and 0.01% Triton X. Each well of a 96-well plate were loaded with 10 µg protein (quantified via Bradford method, against bovine serum albumin (BSA) as standard), 40 µM NADP^+^, 400 µM G6P, 1.22 mM DTNB, 1.25 U/mL G6P-DH and 1.25 U/mL GR. The increasing rate of 5-thio-2-nitrobenzoic acid was measured every 15 s for 30 min at λ = 412 nm with a shaking period of 5 s at 25 °C with a microplate reader. The GSH/GSSG concentration was quantified by interpolation with GSH standards (eight standard concentrations between 0.375–3.0 µM) and related to the used protein concentration. GSH/GSSG content is expressed in ratios of µg to mg of total protein.

### 2.8. Western Blotting of Protein Expression

For the determination of the protein expression, western blotting was done following the instructions for the precast Criterion TGX Stain-Free Gels and the corresponding Trans_Blot Turbo Pack Midi PVDF membranes from Bio-Rad (Feldkirchen, Germany). The cell line samples were collected and lysed with a buffer containing 50 mM Tris (pH 7.4), 100 mM NaCl, 100 mM NaF, 5 mM EDTA, 0.2 mM Na_3_VO_4_, 0.1% Triton-X and freshly added 1% protease inhibitor cocktail (PIC), on ice for 30 min followed by a sonication for 10 min. After a centrifugation step at 18,000× *g* for 10 min at 4 °C the protein concentration was quantified via Bradford method against BSA as standard. Protein samples were stored at −80 °C till usage. For the electrophoretic separation 30 µg of total protein amount were diluted in 22.5 µL deionized water and 7.5 µL 4x Laemmli Buffer containing 0.65 mM 1,4-dithiothreitol, 18.66 µM bromophenol blue, 0.25 mM Tris (pH 6.8), sodium lauryl sulfate 7.5%, and 37.5% glycerol in water. Each slot of the precast gels was loaded with protein samples and after electrophoretic separation blotted onto PVDF-membranes by using the Trans-Blot Turbo System from Bio-Rad. Blots were blocked with 10% non-fat milk powder in Tris buffered saline/tween buffer (TBST) containing 0.02 mM Tris, 0.145 mM NaCl and 0.5% Tween 20 in water for 2 h and incubated with primary antibody dilution (1:1000, or 1:500 for Txr1 and Trx2) in TBST plus 1% BSA over night at 4 °C. After a washing procedure with TBST, blots were incubated with horse radish peroxidase conjugated secondary antibody dilution (1:5000) in TBST plus 1% BSA for 2 h at room temperature. Selected protein bands were detected with Clarity Western ECL Substrate and recorded with an Advanced Fluorescence Imager (INTAS Science Imagining, Göttingen, Germany). As a loading control, total protein normalization was done by using the TGX Stain-Free gel system [28,29].

### 2.9. Determination of IC_50_ Values

For the determination of the anticancer potency of various anticancer drugs on HAP-1 cells, the IC_50_ values were determined with MTT cell viability assay as described previously [30]. Briefly, cells were seeded out in 96-well plates at a density of 5000 cells in 100 µL culture medium and allowed to attach for 24 h. Compounds were dissolved in either *N,N*-dimethylformamide (DMF; for the platinum compound) or dimethyl sulfoxide (DMSO; for all other compounds), then serial diluted at 1000-fold the intended concentration before being diluted 1:500 into culture medium. To each well was added 100 µL test solution, giving a total volume of 200 µL/well with final concentrations of DMF or DMSO of 0.1% (*v/v*). Final concentrations are shown in the figure. After an incubation period of 48 h, 40 µL of a 2.5 mg/mL solution of MTT in Dulbecco’s buffer was added to each well and incubated for 4 h at 37 °C. Then the medium was removed and replaced by 50 µL DMSO, then placed on a plate shaker 5 min to dissolve the formazan crystals. The optical density was measured with a plate reader set at λ = 570 nm; ODs were expressed as ratios to the ODs of the test wells to the OD of the untreated control (T/C%). The results of T/C% were plotted against the concentration of the tested drug on a logarithmic scale. The inhibition of viability to 50% (IC_50_) was calculated via interpolation at 50% with GraphPad Prism Software 6.0.

### 2.10. Determination of ROS-Levels after Peroxide or Anticancer Drug Treatment

For the determination of the ROS-content of cells after incubation with anticancer drugs, a cell cytometer based technique with the intracellular ROS sensor reagent DCF-DA was used as previously described [30]. Briefly, cells were seeded out in a density of 50,000 cells per 3 mL, into a 6-well plate and allowed to attach for 24 h. Then, cells were exposed to anticancer drugs in fractions of the IC_50_ value and incubated for 24 h. After removal of the medium, cells were harvested with trypsin/EDTA and washed with PBS by a centrifugation and resuspension step, then treated with 500 µL of a 20 µM DCF-DA solution in PBS for 30 min in the dark. The staining solution was replaced with 500 µL PBS, followed by flow cytometry determination at λ_excitation_/λ_emission_ = 488/527 nm with a MACSQuant Analyser 10 (Miltenyi Biotec, Teterow, Germany). Debris was excluded in the dot plot of the forward vs. sideward scatter. The mean fluorescence signal of DCF in treated cells was related to the untreated control population.

### 2.11. Statistics

The GraphPad Prism 6.0 program (GraphPad Software, La Jolla, USA) was used for all statistical evaluations. A two-tailed paired t-test was used for the comparison of IC_50_ values of the anticancer drugs and the peroxides in the knockout cell line (KO.HAP-1.GPx1), compared to the parental HAP-1 cell line as well as for comparison of the GSH content in both cell lines. For the determination of varying ROS accumulation, a two-way ANOVA test was performed with the Sidak’s adaption of a multiple comparison.

## 3. Results

### 3.1. Characterization of GPx1 Knockout HAP-1 Cells Relative to the Native Cell Line

**Figure 1:**
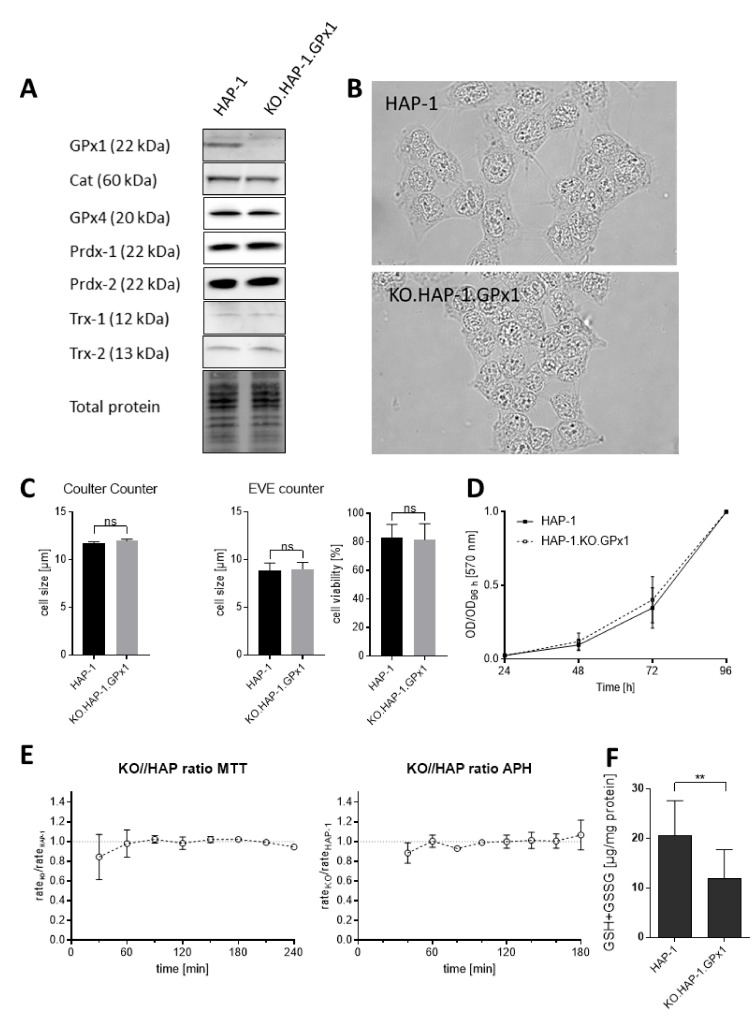

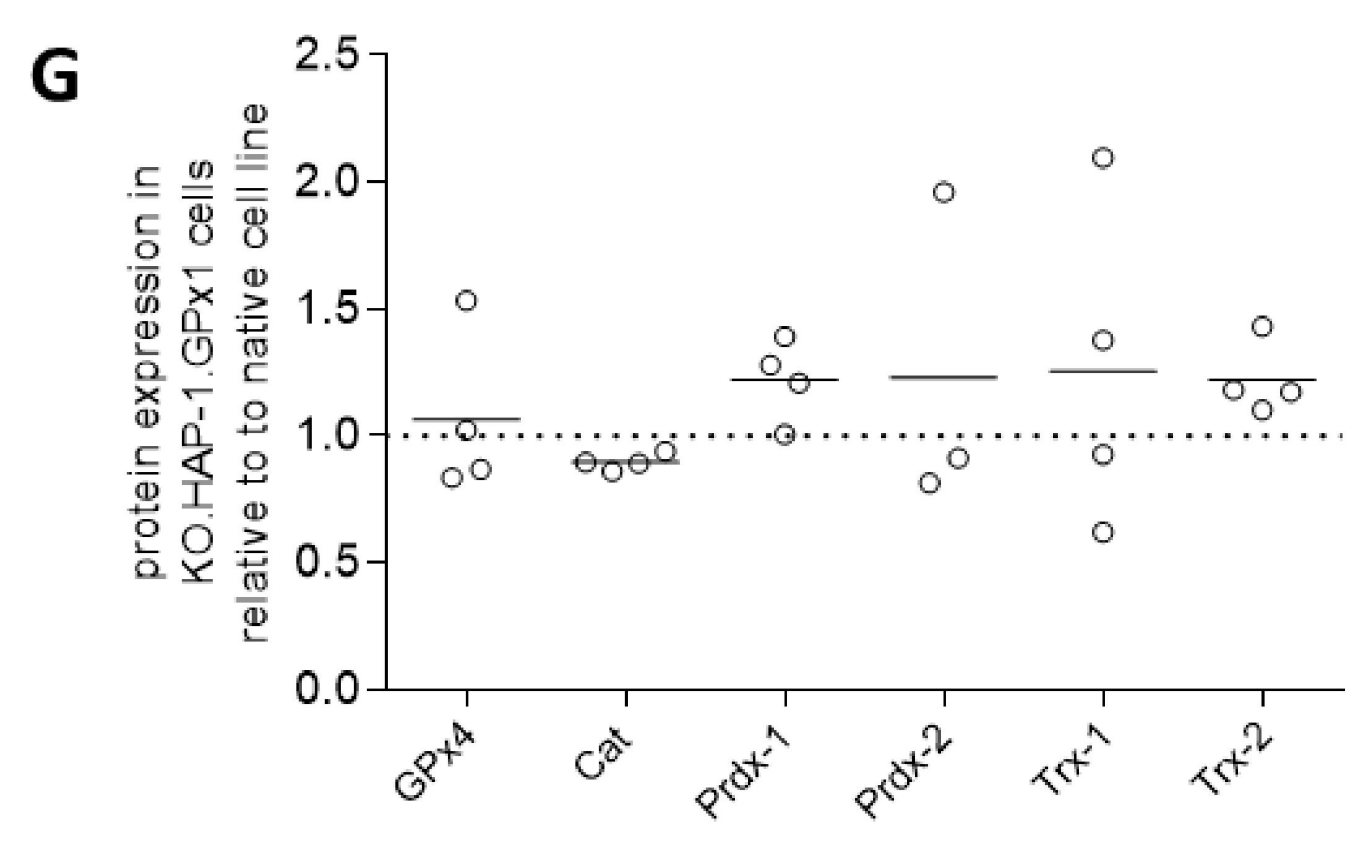
(**A**): Representative western blots of protein expression in parental HAP-1 cells and its GPx1 knockout variant KO.HAP-1.GPx1 for glutathione peroxidase-1 and -4 (GPx1, GPx4), catalase (Cat), peroxiredoxin-1 and -2 (Prdx1, Prdx2) and thioredoxin-1 and -2 (Trx1, Trx2) while total protein normalization was used as a loading control; (**B**): Microscopic images of HAP-1 and KO.HAP-1.GPx1 cells; (**C**): Cell sizes and viability in HAP-1 and knockout cells in suspension as measured by Coulter Counter and EVE counter, respectively, *n* = 3; (**D**): Relative growth of HAP-1 and KO.HAP-1.GPx1 cells as determined via crystal violet proliferation assay over the course of 4 days, *n* = 3; (**E**): Relative conversion rates for the MTT and APH assay in KO.HAP-1.GPx1 cells compared to rates in HAP-1 cells.; *n* = 3 (SD not shown when smaller than symbol height). (**F**): Total glutathione content in cell lysates of HAP-1 cells and in its GPx1 knockout; (**G**): corresponding relative protein expression in knockout cells relative to parental HAP-1 cells. All numerical results show mean ± standard deviations of at least 3 independent experiments. Significance tested by unpaired, two-sided *t*-test; ** *p* < 0.01.

The cell line used in this work was the HAP-1 line, which is a near-haploid cell line derived from the KBM-7 cell line, a line obtained from a chronic myeloid leukaemia (CML) patient [31]. Haploid cells offer a unique model system to study the effects of knocking out single genes, assuring complete removal of the coding protein. A 38 bp deletion in the GPx1 gene was performed by Horizon Discovery via CRISPR-Cas9 technology; sequencing of the clone (KO.HAP-1.GPx1) confirmed the deletion. (The corresponding knockout sequences can be found in the vendor’s information in Figures S1 and S2 in the supplemental material). The success of the GPx1 knockout was further confirmed by western blotting for the GPx1 protein and shown in Figure 1A. The parental HAP-1 cell line had a moderate GPx1 expression whereas in KO.HAP-1.GPx1 cells loss of the GPx1 band was observed. Thus, the knockout procedure was successful.

HAP-1 and KO.HAP-1.GPx1 were also characterized by microscopic appearance, size, doubling times, cell viability, metabolic rates, GSH + GSSG content and expression of other antioxidative enzymes (Figure 1). The microscopic images of both cell lines are shown in Figure 1B; the nuclei of the two lines appear similar but the cytoplasm of the knockout line seemed somewhat reduced in volume. Thus, we measured the diameter of the cell lines in suspension by two different sizing methods: (1) Coulter Counter and (2) optical image analysis by using an EVE^TM^ automated cell counter. While the sizes of the cells varied depending on the method used, both methods revealed no differences in the diameters of the two cell lines (Figure 1C). Moreover, the live/dead ratios based on Trypan blue staining were identical for both cell lines.

We also characterized the two cell lines with regards to their doubling times because doubling times of cancer cells can be associated with their sensitivities to anticancer drugs. No differences in growth rates were found (Figure 1D), with doubling times of ca. 15 h for both lines. Next, cellular metabolic rates were assessed by two different methods: reduction rates of MTT and enzymatic activity of acidic phosphatase (APH). No differences were found between the rates of cellular MTT reduction and APH hydrolysis over a 240 and 180 h time period, respectively. (Figure 1E) ATP content of both cell lines was also determined and found to be nearly identical (data not shown), again indicating similar levels of cellular metabolism.

Loss of GPx1 activity might be expected to be compensated by increases in other antioxidative processes in the knockout cell line. Glutathione (GSH) is the most abundant non-protein thiol in cells and an important non-enzymatic defense against oxidative stress. It is also a substrate for GPx1, with two GSH equivalents being consumed for each peroxide reduced. The total GSH + GSSG content of both HAP-1 cell lines was measured by an enzymatic assay, based on the oxidation of GSH to the disulfide GSSG by reaction with DTNB followed by its recycling back to GSH via a NADPH/glutathione reductase (GR) and glucose-6-phosphate (G6P)/glucose-6-phosphate dehydrogenase (G6P-DH) system [27]. Surprisingly, the GSH + GSSG content was found to be twice as great in the native cell line compared to the knockout variant, suggesting that the loss of GPx1 activity results in a significant reduced cellular dependency on glutathione. (Figure 1F) To assess whether the GPx1 knockout cell line had adapted other antioxidative mechanisms, western blotting was done to monitor the expression of other antioxidative enzymes, i.e., GPx4, catalase (Cat), the peroxiredoxin-1 and -2 (Prdx1, Prdx2) and thioredoxin-1 and -2 (Trx1, Trx2). Representative western blots are shown in Figure 1A as well as the plotted relative protein expression ratios in Figure 1G Protein expression was detectable for all these enzymes. In contrast to GPx1, no difference in the expression of GPx4 was found in KO.HAP-1.GPx1 relative to parental line. For Prdx1 and Trx1 a slight but non-significant increase in protein expression was observed in KO.HAP-1.GPx1 while for Cat a very slight but non-significant decrease in protein expression was noted in the knockout line. Likewise, for Prdx2 and Trx-2, no adapted regulation in the knockout cell line was found.

### 3.2. Effect of GPx1 Knockout in HAP-1 Cells on Toxicity of Peroxides and Anticancer Drugs

**Figure 2:**
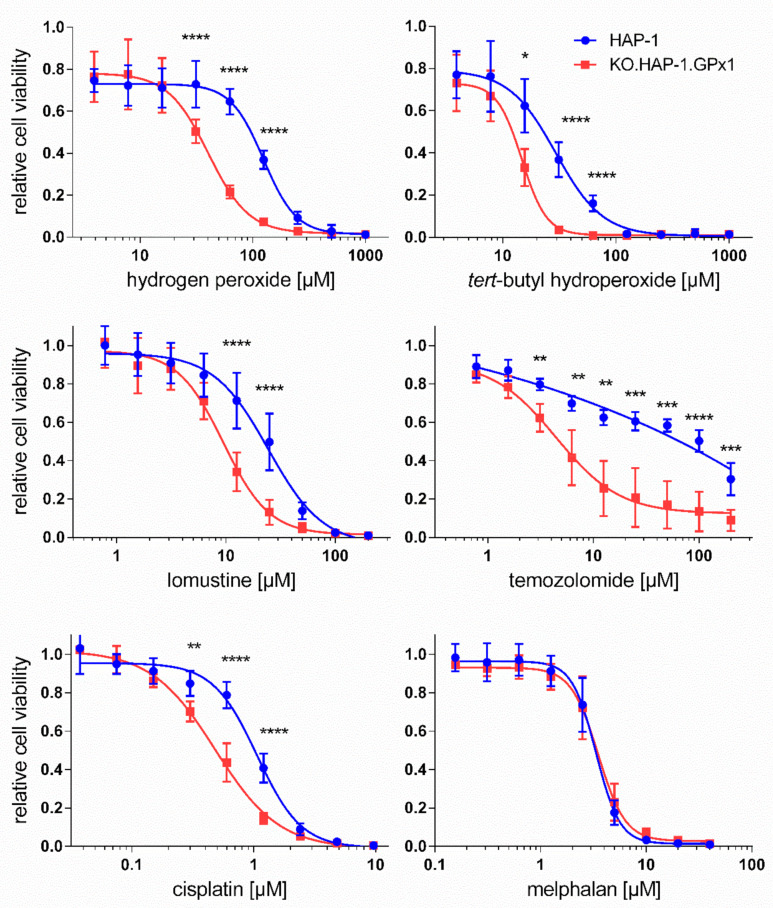
Dose-response curves for the inhibition of cell viability by peroxides and several anticancer drugs in HAP-1 (blue) and KO.HAP-1.GPx1 cell line (red) after 48 h incubation time, as determined by the), 3-(4,5-dimethylthiazol-2-yl)-2,5-diphenyltetrazolium bromide (MTT) assay. All results show mean ± standard deviation of at least four independent experiments. Significance tested by unpaired, two-sided *t*-test: * *p* < 0.05, ** *p* < 0.01, *** *p* < 0.001, **** *p* < 0.0001.

The MTT viability assay was then used to evaluate the effect of a GPx1 knockout in cancer cells towards the cytotoxicity of anticancer drugs. Treatments with two peroxides, H_2_O_2_ and *tert*-butyl hydroperoxide were also performed as positive controls. Representative dose-response curves over more than 100-fold concentration ranges are shown in Figure 2. For these studies, the IC_50_ values of each compound was determined in the parental and in the GPx1 knockout HAP-1 variant, KO.HAP-1.GPx1. The knockout-index (KOI) is the quotient of the IC_50_ value of the knockout cell line divided by the IC_50_ of the parental cell line. A KOI value of one indicates that the knockout of GPx1 had no effect on the cytotoxic potency of the compound. Is the KOI less than one, then the toxicity of drug is enhanced, whereas a KOI greater than one indicates a diminished potency of the anticancer drug in knockout cells. Table 1 shows the IC_50_ values of tested anticancer drugs and peroxides for the viability inhibition in both cell lines along with their corresponding KOI values.

Hydrogen peroxide and *tert*-butyl hydroperoxide were toxic to the HAP-1 cell line with IC_50_ values of 92.4 and 22.1 µM, respectively (Table 1). As expected, the knockout cell line showed a statistically significant increased toxicity to both peroxides, with IC_50_ values of 29.6 and 10.7 µM, respectively. The KOI values of 0.32 and 0.48 represent a two to threefold higher sensitivity of knockout cells towards peroxide toxicity compared to the parental cells. These observations are reminiscent of experiments done with GPx1 knockout mice exposed to diquat, an herbicide known to cause sever oxidative stress in liver cells; all mice lacking the gene died within hours of injection of a single dose of diquat whereas none of the wild-type mice were affected even after seven days [32].

The DNA alkylating agents chlorambucil, melphalan and thiotepa had similar IC_50_ values in both cell lines, with IC_50_ ranging between 3.4 and 7.0 µM. On the other hand, two other DNA alkylating compounds lomustine and temozolomide showed a significant increased cytotoxicity in GPx1 knockout cells in comparison to parental cell line. For lomustine and temozolomide, KOIs of 0.43 and 0.09 were determined, respectively. Thus, temozolomide is ca. eleven times more toxic in GPx1 knockout cells than in parental HAP-1 cells. The antimetabolite methotrexate (IC_50_ ~ 65 nM) and the spindle poisons vinblastine (IC_50_ ~ 1.65 nM), paclitaxel (IC_50_ ~ 2.75 nM), camptothecin (IC_50_ ~ 3.85 nM), colchicine (IC_50_ ~ 13.5 nM) and podophyllotoxin (IC_50_ ~ 20 nM) all showed not differences in IC_50_ values between the two cell lines. The proteasome inhibitor bortezomib and the anthracycline doxorubicin, a drug known to be redox active, gave a trend to increasing toxicity in the knockout cell line with KOIs around 0.84, but these were not significant. For the DNA platinating agent cisplatin and its prodrug form carboplatin, significant enhancements in cytotoxicity in the knockout cell line were also found; cisplatin toxicity increased from an IC_50_ value of 1.06 µM in parental cells to 0.52 µM in knockout cell line with a KOI of 0.49 while for the cisplatin prodrug carboplatin a KOI of 0.83 was determined. For oxaliplatin a non-significant trend to increased potency in knockout cell line was detectable, with a KOI of 0.71.

### 3.3. Determination of ROS Accumulation Induced by Peroxides, Cisplatin, Lomustine and Temozolomide in HAP-1 Cells and Their GPx1 Knockout Strain

**Figure 3:**
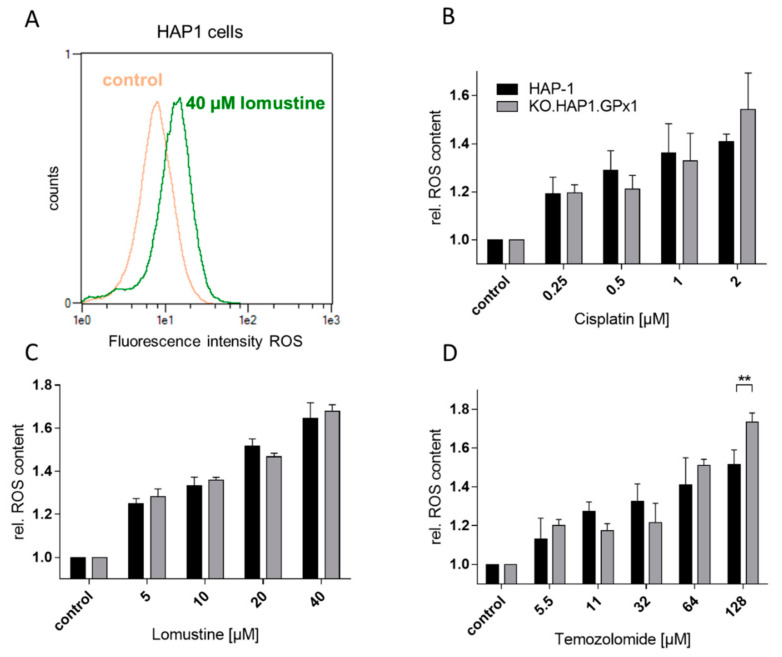
(**A**). Representative histogram showing flow cytometry results from the 2’,7’-dichlorofluorescine diacetate (DCF-DA) assay for ROS levels after a 24 h incubation with 40 µM lomustine in HAP-1 cells; (**B**–**D**). Relative reactive oxygen species (ROS) levels in HAP 1 cells and the GPx1 knockout variant after incubation with either cisplatin (**B**), lomustine (**C**) or temozolomide (**D**) (at the reported concentrations) for 24 h at various concentrations (mean ± standard deviation, *n* = 3; ** *p* < 0.01).

To assess whether the increased anticancer potency of some of the anticancer drugs in the GPx1 knockout cells was due to oxidative stress in the cells, the intracellular ROS levels were measured by a flow cytometric method with the ROS sensitive fluorescent dye 2’,7’-dichlorodihydrofluorescein diacetate (DCF-DA). DCF-DA is widely used for measuring oxidative stress in cells, being particularly sensitive to H_2_O_2_ but other cellular oxidants can also bring about fluorescence [33]. Only the three anticancer drugs were investigated that showed a highly significant difference in IC_50_ values between knockout and native lines. Both cell lines were incubated with either cisplatin, lomustine or temozolomide for 24 h at concentrations above and below their IC_50_ values (Figure 3). A representative flow cytometric histogram is shown in Figure 3A. Figure 3B–D summarize the effects of increasing drug concentration on ROS content in HAP-1 and KO.HAP-1.GPX1 cells. Interestingly, significant concentration-dependent increases in ROS levels were detected for every drug compared to the untreated control, but no differences in ROS accumulation between the GPx1 knockout and native cell lines were noted, with one exception for temozolomide at the highest concentration.

## 4. Discussion

The aim of this work was to assess the influence of the key antioxidative enzyme GPx1 on anticancer drug cytotoxicity. In this proof-of-concept study, we used a GPx1 knockout cell line, obtained by a CRISPR-Cas9 deletion of the only GPX1 gene in HAP-1 haploid human cancer cells, to analyze for differences in potency of widely used anticancer drugs to the parental line. Characterization of both cell lines showed slight differences in the morphology but no differences in size, doubling-time, viability or rates of metabolism. Our next focus was on the expression of various redox associated enzymes and intracellular GSH + GSSG content. It was anticipated that the knockout cell line would adapt to the loss of GPx1 function by increasing the expression of other antioxidant enzymes and/or GSH + GSSG. However, with the exception of a complete loss of GPx1 expression, we found no other significant alterations in the knockout cell line with respect to catalase, GPx4, peroxiredoxin-1 and -2 and thioredoxin-1 and -2. This suggests that under normal growth conditions, GPx1 is not essential for maintenance of a healthy redox balance. However, against our intuition we found a significant decrease in GSH + GSSG content in the knockout cells relative to the native cell line, suggesting a more complicated relationship between GPx1 and glutathione metabolism.

Pursuant to our question of the influence of GPx1 expression on anticancer drug sensitivity, the cytotoxic potency of two peroxides and sixteen anticancer were examined in HAP-1 and KO.HAP-1.GPx1 cells. Not surprisingly, we detected knockout indices below one for both peroxides. The association between loss of GPx1 function with an increased sensitivity to peroxides has long been recognized [34]. Importantly, we identified a significant enhancement in potency for the platinum-based agents cisplatin and carboplatin when GPx1 is deleted. For the third platinum anticancer drug oxaliplatin, we also observed a trend in this direction but it was not significant. Likewise, the DNA alkylating agents lomustine (nitrosourea) and temozolomide (a methylating agent) also showed an increase in potency upon deletion of GPx1, whereby the increase was particularly apparent for temozolomide with a KOI of just 0.09. Interestingly, the other three DNA alkylating agents, melphalan, chlorambucil (nitrogen mustards) and thiotepa showed no such behavior, as well as the remaining anticancer drugs, including the spindle poisons vinblastine, paclitaxel, colchicine and podophyllotoxin, the topoisomerase I inhibitor camptothecin, the proteasome inhibitor bortezomid, the DNA intercalator doxorubicin, and the dihydrofolic acid reductase inhibitor methotrexate.

Chen et al. recently showed that an increased GPx1 expression leads to resistance to cisplatin in non-small cell lung cancer cells whereas a downregulation via silencer RNA sensitizes tumor cells towards the drug [12]. These findings are consistent with ours for cisplatin and carboplatin (a cisplatin prodrug) in HAP-1 cells. Moreover, an inverse association between GPx1 expression and temozolomide potency in two glioblastoma cell lines has recently been described [35]; our results with the HAP-1 cell lines support this finding. Several other studies have reported the development of resistance to anticancer drugs concomitant with an upregulation of GPx1 [13,36,37]. However, to the best of our knowledge this is the first report linking the potentiation of lomustine cytotoxic activity to the lost expression of GPx1 in cancer cells.

On the basis of the varying potency of the anticancer drugs in the GPx1 positive and negative cell lines, we expected differences in ROS accumulation after incubation with cisplatin, lomustine and temozolomide. It is known that an incubation of these drugs can bring about an enhancement in ROS levels, which is connected to the anticancer drug potency [38,39,40]. We also observed a concentration dependent increase in intracellular ROS levels with all three drugs. However, contrary to our expectations, no differences in ROS levels between the two cell lines were observed except at the highest concentration of lomustine, for which we also observed the strongest enhancement in antiproliferative activity. This suggests that the increasing potencies of cisplatin, lomustine and temozolomide may also be due to secondary effects of the GPx1 knockout, such as the lowering of intracellular GSH + GSSG levels upon GPx1 deletion, which we also observed. Future research is needed to understand the effects of these three anticancer drugs on GSH levels in the two cell lines.

In conclusion, it could be shown that GPx1 is not required for cell viability under normal cell culture conditions; this finding is consistent with earlier studies with knockout mice. Thus, therapeutic inhibition of GPx1 might not be expected to be toxic to cells under normal conditions. For the first time an association has been shown between GPx1 expression and anticancer potency of lomustine. Finally, inhibition of GPx1 would appear to be a worthwhile therapeutic goal to potentiate the cytotoxic activity of a number of anticancer drugs. Recently identified pentathiepins as potent inhibitors of GPx1 are being investigated with this strategy in mind [30].

## Figures and Tables

**Table 1:** IC_50_ values for inhibition of cell viability by anticancer drugs and peroxides in HAP-1 and KO.HAP-1.GPx1 cell line and corresponding KOI values [mean ± SD; *n* ≥ 3]. *p*-Values are the levels of significance between the IC_50_ values for the two cell lines as tested with a two-sided, paired *t*-test; only significant *p*-values are shown.

	HAP-1	KO.HAP-1.GPx1	KOI	*p*
Hydrogen peroxide	92.4 ± 7.3 µM	29.6 ± 7.0 µM	0.32	0.0001
*tert*-Butyl hydroperoxide	22.1 ± 7.7 µM	10.7 ± 3.2 µM	0.48	0.05
Melphalan	3.44 ± 0.56 µM	3.55 ± 0.73 µM	1.03	-
Chlorambucil	6.96 ± 1.78 µM	7.01 ± 1.82 µM	1.01	-
Thiotepa	6.21 ± 1.06 µM	6.34 ± 0.56 µM	1.02	-
Lomustine	23.0 ± 6.9 µM	9.84 ± 1.94 µM	0.43	0.005
Temozolomide	63.9 ± 21.7 µM	5.53 ± 2.42 µM	0.09	0.005
Vinblastine	1.58 ± 0.33 nM	1.74 ± 0.50 nM	1.10	-
Paclitaxel	2.82 ± 0.42 nM	2.52 ± 0.53 nM	0.90	-
Camptothecin	3.88 ± 0.09 nM	3.83 ± 0.43 nM	0.99	-
Colchicine	13.0 ± 2.6 nM	14.0 ± 4.4 nM	1.08	-
Podophyllotoxin	19.5 ± 1.0 nM	20.1 ± 1.4 nM	1.03	-
Bortezomib	13.3 ± 4.8 nM	11.4 ± 2.3 nM	0.85	-
Doxorubicin	24.4 ± 7.4 nM	21.5 ± 4.4 nM	0.82	-
Methotrexate	64.7 ± 5.9 nM	64.8 ± 4.4 nM	1.00	-
Cisplatin	1.06 ± 0.13 µM	0.52 ± 0.07 µM	0.49	0.0005
Carboplatin	29.6 ± 18.6 µM	24.4 ± 16.0 µM	0.83	0.05
Oxaliplatin	0.94 ± 0.29 µM	0.67 ± 0.17 µM	0.71	-

## Supplementary Materials

The following are available online at https://www.mdpi.com/2076-3921/9/12/1300/s1, Figure S1: Position of the frameshift mutation within the GPx1 gene, Figure S2: Sequencing result of clone, mapped on NM_000581, Figure S3: Representative western blot of human GPx1 in various cell lines; corresponding signals in HAP-1 and KO.HAP-1.GPx1 cells are tagged. Positive GPx1 control was done with bovine GPx, Figure S4: Representative dot plots from flow cytometric analysis of the Annexin V-FITC/PI Assay in untreated HAP-1 and KO.HAP-1.GPx1 cells determining background apoptosis, Figure S5: Representative western blot of human GPx4 in HAP-1 and KO.HAP-1.GPx1 cells, Figure S6: Representative western blot of human catalase in various cell lines; corresponding signals in HAP-1 and KO.HAP-1.GPx1 are tagged, Figure S7: Representative western blot of Prx1 and Prx2 in HAP-1 and KO.HAP-1.GPx1 cells, Figure S8: Representative western blot of human Trx1 in HAP-1 and KO.HAP-1.GPx1 cells, Figure S9: Representative western blot of Trx2 in HAP-1 and KO.HAP-1.GPx1 cells.

## Abbreviations

APH	Acidic phosphatase
BSA	Bovine serum albumin
Cat	Catalase
CDF-DA	2′,7′-dichlorodihydrofluorescein diacetate
DTNB	Ellman’s reagent, 5,5-Dithiobis(2-nitrobenzoic acid)
FCS	Fetal calf serum
GSH	Reduced glutathione
GSSG	Glutathione disulfide
GSH + GSSG	Total glutathione
GPx	Glutathione peroxidase
GR	Glutathione reductase
G6P	Glucose-6-phosphate
G6P-DH	Glucose-6-phosphat dehydrogenase
KOI	Knockout index
LPO	Lipid peroxidation
MTT	3-(4,5-Dimethylthiazol-2-yl)-2,5-diphenyltetrazolium bromide
PBS	Phosphate buffered saline
Prdx	Peroxiredoxin
ROS	Reactive oxygen species
TrxR	Thioredoxin reductase
