# Supplementary material for: The Effect of Glutathione Peroxidase-1 Knockout on Anticancer Drug Sensitivities and Reactive Oxygen Species in Haploid HAP-1 Cells
Source: Antioxidants (Basel). 2020 Dec 18;9(12):1300. doi: 10.3390/antiox9121300 (PMC7766971; doi:10.3390/antiox9121300)
Supplement: Supplementary file 1 [file antioxidants-09-01300-s001.pdf]

The Effect of Glutathione Peroxidase-1knockout on Anticancer Drug Sensitivities and the Accumulation of Reactive Oxygen Species in Hap-1 Human Cancer Cells

Supporting information

Steven Behnisch-Cornwell, Lisa Wolff, Patrick J. Bednarski

List of Figures:

Figure S1. Position of the frameshift mutation within the GPx1 gene.

Figure S2. Sequencing result of clone, mapped on NM_000581.

Figure S3. Representative western blot of human GPx1 in various cell lines; corresponding signals in HAP-1 and KO.HAP-1.GPx1 cells are tagged. Positive GPx1 control was done with bovine GPx.

Figure S4. Representative dot plots from flow cytometric analysis of the Annexin V-FITC/PI Assay in untreated HAP-1 and KO.HAP-1.GPx1 cells determining background apoptosis.

Figure S5. Representative western blot of human GPx4 in HAP-1 and KO.HAP-1.GPx1 cells.

Figure S6. Representative western blot of human catalase in various cell lines; corresponding signals in HAP-1 and KO.HAP-1.GPx1 are tagged.

Figure S7. Representative western blot of Prx1 and Prx2 in HAP-1 and KO.HAP-1.GPx1 cells.

Figure S8. Representative western blot of human Trx1 in HAP-1 and KO.HAP-1.GPx1 cells.

Figure S9. Representative western blot of Trx2 in HAP-1 and KO.HAP-1.GPx1 cells.

Guide RNA sequence: TAAGTAGTACCTTGCCCCGC Clone: 3261-10

Mutation: 38bp deletion in exon 1, causing frameshift

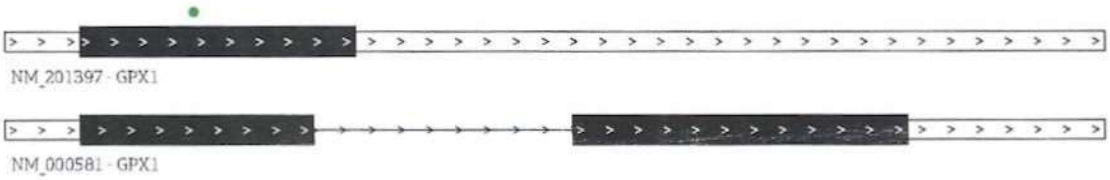

Figure S1. Position of the frameshift mutation within the GPx1 gene.

```
15 CTAGCGGCGGCGGCGGCGGCGGCCCAGTCGGTGTATGCCTTCTCGGCGCGCCCGCTGGCC
   ||||||||||||||||||||   ||||||||||||||||||||||||||||||||||||
   CTAGCGGCGGCGGCGGCGGC---CCAGTCGGTGTATGCCTTCTCGGCGCGCCCGCTGGCC
   LeuAlaAlaAlaAlaAlaAlaAlaGlnSerValTyrAlaPheSerAlaArgProLeuAla

   GGCGGGGAGCCTGTGAGC 93
   ||||||||||||||||||
   GGCGGGGAGCCTGTGAGC
   GlyGlyGluProValSer
```

Figure s2. Sequencing result of clone, mapped on NM_000581.

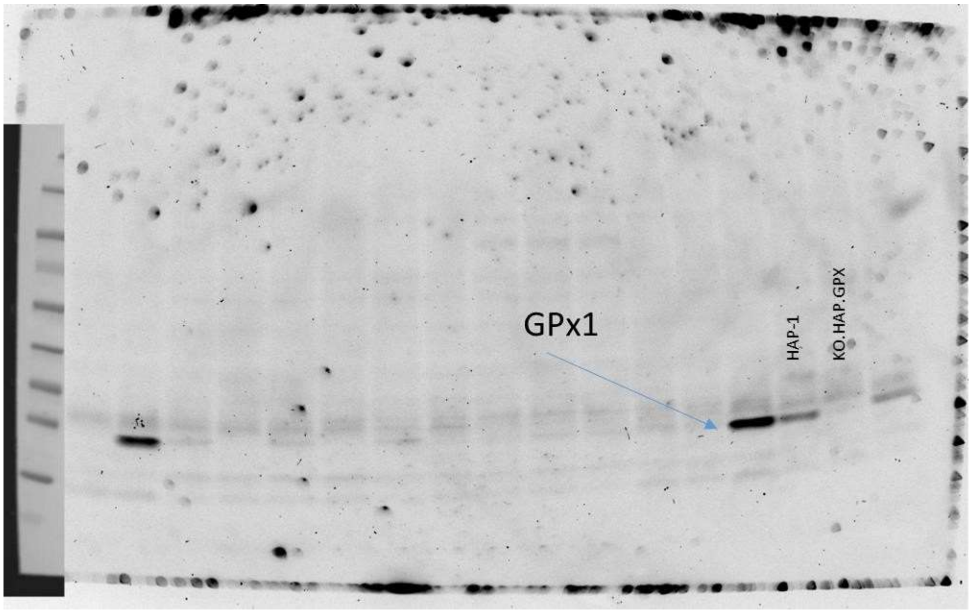

Figure S3. Representative western blot of GPx1 in various cancer cell lines. The corresponding lanes for HAP-1 and KO.HAP-1.GPx1 cells are tagged. Positive control was run with purified bovine GPx1.

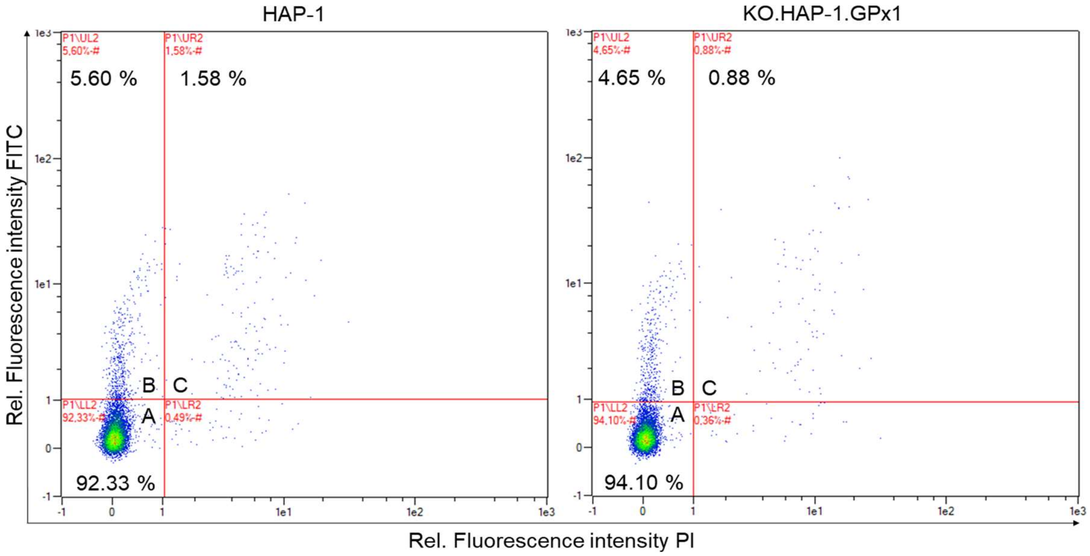

Figure S4. Representative dot plots from flow cytometric analysis of the Annexin V-FITC/PI Assay in untreated HAP-1 and KO.HAP-1.GPx1 cells determining background apoptosis. Displayed on x-axis: relative fluorescence intensity of propidium iodide (PI). Displayed on y-axis: relative fluorescence intensity of fluorescein isothiocyanate (FITC). Analysis quadrants: **A**) viable cells (FITC/PI negative) **B**) early apoptotic cells (FITC positive/PI negative) **C**) late apoptotic cells (FITC/PI positive).

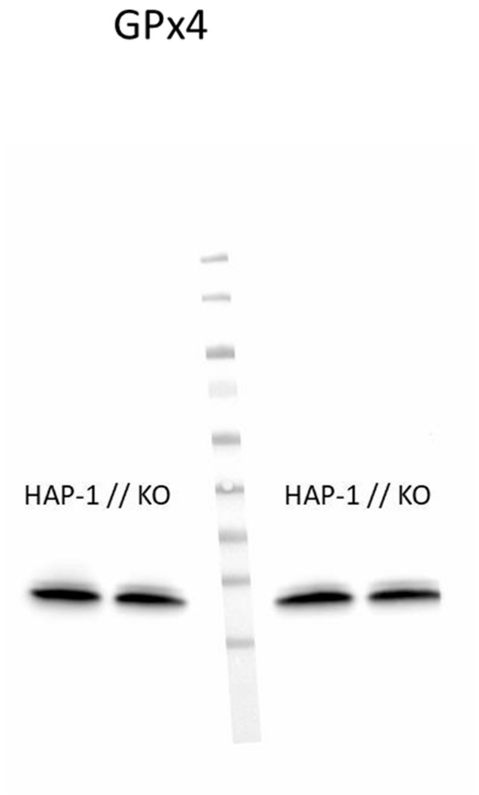

Figure S5. Representative western blot of GPx4 in HAP-1 and KO.HAP-1.GPx1 cells.

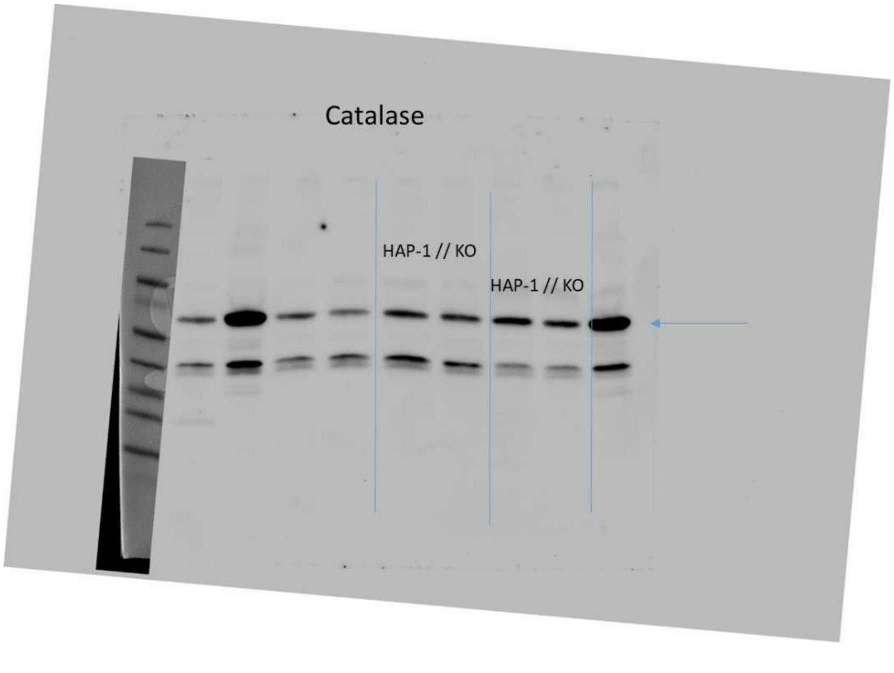

Figure S6. Representative western blot of catalase in various cell lines; corresponding signals in HAP- 1 and KO.HAP-1.GPx1 are tagged.

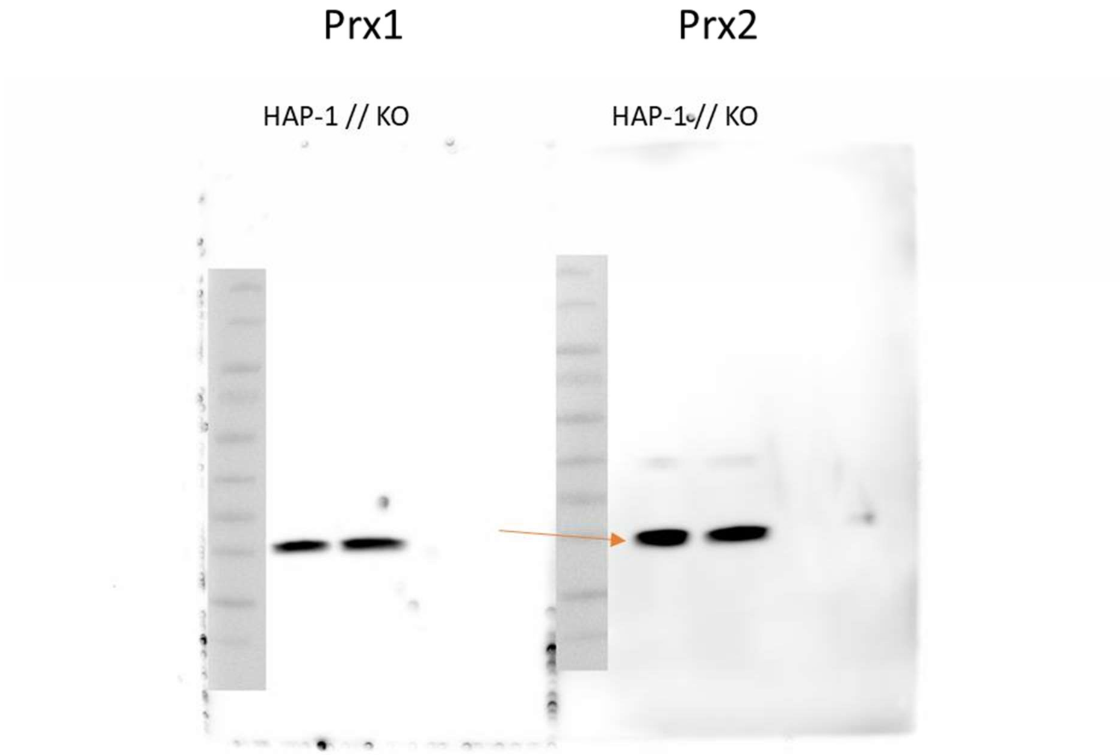

Figure S7. Representative western blot of Prx1 and Prx2 in HAP-1 and KO.HAP-1.GPx1 cells.

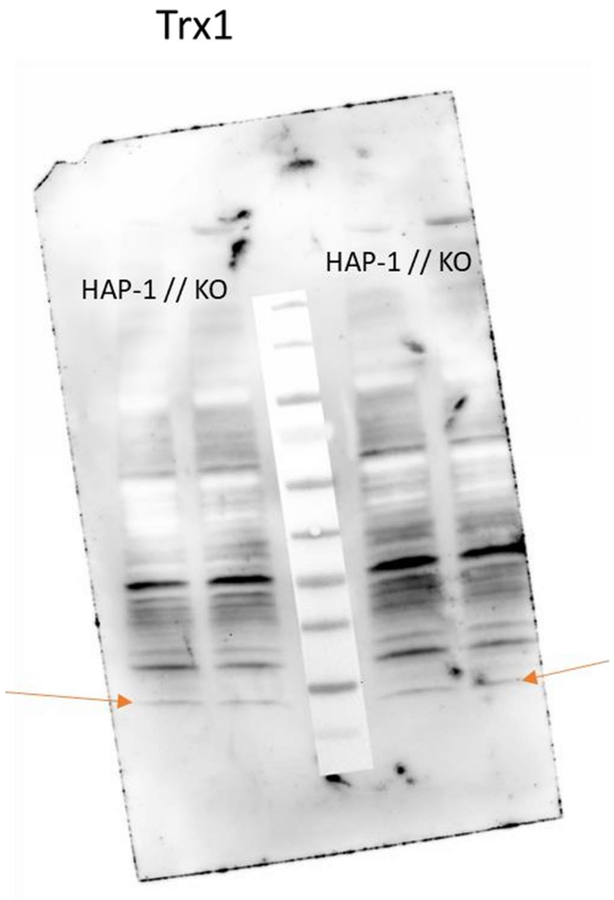

Figure S8. Representative western blot of Trx1 in HAP-1 and KO.HAP-1.GPx1 cells.

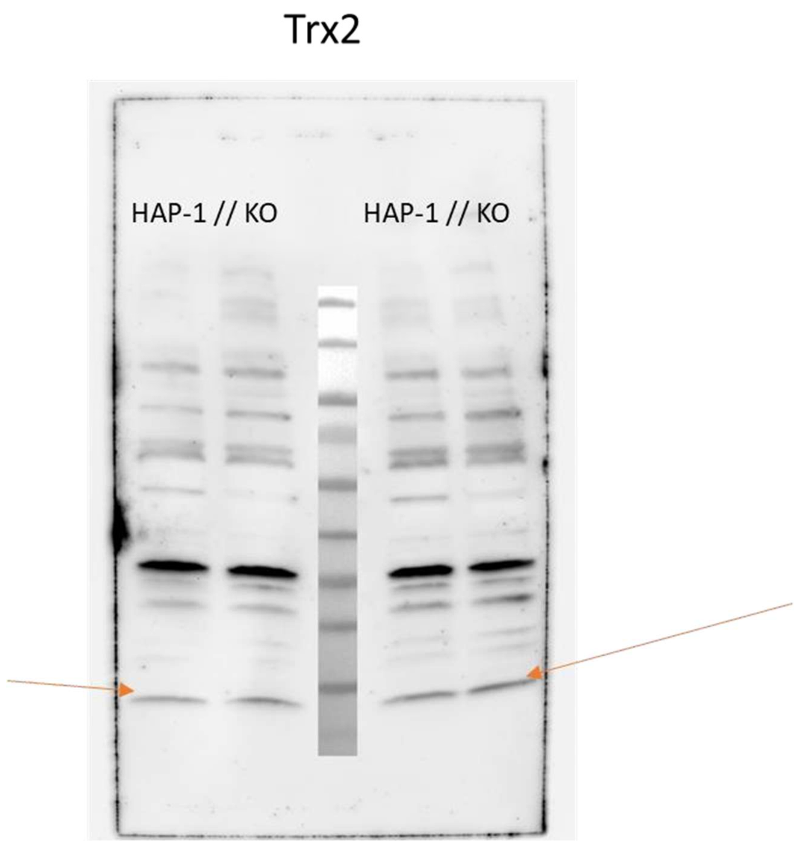

Figure S9. Representative western blot of Trx2 in HAP-1 and KO.HAP-1.GPx1 cells.
